# Analysis of pathogenic factors on the death rate of sepsis patients

**DOI:** 10.1371/journal.pone.0287254

**Published:** 2023-12-14

**Authors:** Luwei Ye, Mei Feng, Qingran Lin, Fang Li, Jun Lyu

**Affiliations:** 1 Intensive Care Unit, The First Affiliated Hospital of Jinan University, Guangzhou, Guangdong Province, China; 2 Department Of Nursing, The First Affiliated Hospital of Jinan University, Guangzhou, Guangdong Province, China; Pikeville Medical Center, UNITED STATES

## Abstract

**Background:**

The Surviving Sepsis Campaign (SSC) believed that early identification of septic shock, aggressive fluid resuscitation and maintenance of effective perfusion pressure should be carried out. However, some of the current research focused on a single death factor for sepsis patients, based on a limited sample, and the research results of the relationship between comorbidities and sepsis related death also have some controversies.

**Method:**

Therefore, our study used data from a large sample of 9,544 sepsis patients aged 18–85 obtained from the MIMIC-IV database, to explore the risk factors of death in patients with sepsis. We used the general clinical information, organ dysfunction scores, and comorbidities to analyze the independent risk factors for death of these patients.

**Results:**

The death group had significantly higher organ dysfunction scores, lower BMI, lower body temperature, faster heart rate and lower urine-output. Among the comorbidities, patients suffering from congestive heart failure and liver disease had a higher mortality rate.

**Conclusion:**

This study helps to identify sepsis early, based on a comprehensive evaluation of a patient’s basic information, organ dysfunction scores and comorbidities, and this methodology could be used for actual clinical diagnosis in hospitals.

## 1. Introduction

Sepsis is the leading cause of patient death in hospitals [1], and about 3 million patients who survive sepsis have cognitive impairment. Sepsis is a life-threatening organ dysfunction caused by a dysregulated body response to infection, which can lead to multiple organ failure and, in severe cases, death [2]. According to epidemiological surveys, about 150,000 people die of sepsis in Europe every year [3], while nearly 500 patients die of sepsis every day in the United States [4]. In 2020, Rudd et al. [5] described that sepsis affects nearly 50 million people worldwide every year, with mortality rates ranging from 5% to 40%. It is one of the global public health burdens. A survey by Angus et al. [6] found that the average cost of severe sepsis in the United States is $22,000 per case, for an annual cost as high as $16.7 billion for the whole country. With the increase of births around the world, the aging of the population, the increase of invasive procedures, high-risk surgery, severe trauma, organ transplantation, radiotherapy and chemotherapy, coupled with factors such as pathogen changes and rising antibiotic resistance, the incidence of sepsis is increasing year by year [7]. Sepsis is currently considered to be a complex and diverse pathological process, characterized by a high degree of variability. Its clinical course is mainly affected by the type of infection, genetic factors, treatment timing, treatment methods and other uncertain factors [8]. Despite the rapid recent development and progress of anti-infective treatment regimens and organ function support technologies, mortality due to sepsis remains high [9]. The choice of treatment strategy depends on the identification of the characteristics of critically ill patients and appropriate risk stratification. Early diagnosis and identification of high-risk patients are two key factors in determining the treatment strategy and clinical course [10]. If sepsis is not treated effectively in timely manner, it may progress to severe sepsis, septic shock or even to multiple organ dysfunction syndrome. However, it is often difficult to accurately determine the best treatment for a variety of reasons [11].

Most current studies focus on a single factor that causes the death of patients with sepsis, and only a few studies have examined more comprehensive information about the patients, or looked at the impact of comorbidities on patient mortality. However, some of these studies based on a sepsis patients record from a single hospital generally had a small sample size, therefore, the conclusions were sometimes slightly biased. We therefore used data from 9,544 sepsis patients to conduct a more comprehensive analysis and aims to find the correlative factors of sepsis mortality.

## 2. Materials and methods

### 2.1. Database

This research did not involve any human participants, and did not involve any minors. All data in this study come from the MIMIC-IV database. MIMIC-IV is a multi-parameter database jointly constructed by MIT Computer Physiology Laboratory and Beth Israel Deaconess Medical Center in Boston, and it is a structured single-center critical care database. The MIMIC-IV database includes health information data on approximately more than 250,000 electronic admissions of unidentified patients from 2008–2019. The data includes demographics, vital signs, laboratory tests, and medication regimens. The database has the advantages of large sample size, comprehensive data, long-term patient tracking, and is free to use, which provides a rich resource for intensive care research [12]. The data used in this study were from the latest version, MIMIC-IV 1.0, released in March 2021. After completing the online course from the National Institutes of Health, and passing the examination for the protection of human study participants, we were qualified to use the MIMIC-IV database (Luwei Ye and Mei Feng. Record ID: 46482390).

Variables extracted from the MIMIC IV database for this research included general clinical information, comorbidities, and severity scores. The general clinical information includes age, gender, heart rate, respiratory rate, height, weight, temperature, and urine output in the first 24 hours after admission. We analyzed the accuracy of five commonly used scoring systems for predicting mortality in patients with sepsis, including the Sequential Organ Failure Assessment (SOFA), Logistic Organ Dysfunction System (LODS), Systemic Inflammatory Response Syndrome (SIRS), Oxford Acute Severity of Illness Score (OASIS) and Acute Physiology and Chronic Health Evaluation III (APACHE III). Comorbidities were also analyzed in this research. These included myocardial infarction, congestive heart failure, peripheral vascular disease, cerebrovascular disease, dementia, chronic pulmonary disease, rheumatic disease, peptic ulcer disease, liver disease, diabetes, paraplegia and renal disease.

### 2.2. Data filtering

The following requirements need to be satisfied when choosing data:

1. Aged between 18 and 85 at the time of hospitalization;
2. Meet the diagnostic criteria of Sepsis 3.0;
3. Hospitalization time > 24 hours

Exclusionary criteria:

1. Patients admitted to ICU with neurological diseases, severe chronic complications, subarachnoid hemorrhage, cerebral hemorrhage, stroke, tumor, and cardiac and respiratory arrest that affect the thermoregulatory center;
2. Pregnancy and maternity;
3. Voluntary discharge by the patient or a patient who discontinues treatment.

After determining the stay ID of the study population, we extracted their relevant information using Structured Query Language (SQL) Programming by Navicat Premium 11.2.7.0. To assess the obesity status of patients, their BMI was obtained based on height and weight data. After a preliminary analysis, mild and severe liver disease was seen to have similar effects on patient mortality, so we combined data for these two diseases under the column ‘Liver disease’.

### 2.3. Data analysis

Statistical analysis was performed using SPSS 26.0 (IBM). For continuous variables that followed a normal distribution, we used mean and standard deviation to describe them, and conducted two independent sample t-tests to compare between groups. For continuous variables that did not follow a normal distribution, we used median and quartiles to describe them and conducted Mann-Whitney U-tests to compare between groups. For categorical variables, we used frequency and percentage to describe them, and conducted chi-square tests to compare between groups. For ordinal variables, we used Mann-Whitney U-tests to compare between groups. Multiple logistic regression analysis was used to determine the factors impacting mortality. A significance level of P < 0.05 was used to indicate statistical significance of differences. Only significantly correlated dependent variables were further analyzed in this research.

The data for these 9,544 patients were extracted from the MIMIC database, allowing us to analyze the influence of different general basic physical factors of the sepsis patients on their death rate in hospitals. We also analyzed the relationship between comorbidities and the death rate. The results are expected to inform death prevention and treatment efforts for early clinical treatment in hospitals.

## 3. Results

### 3.1. Organ dysfunction scoring systems

We analyzed the accuracy of five commonly used organ dysfunction scoring systems for predicting mortality in patients with sepsis, including SOFA, LODS, SIRS, OASIS and APACHE III (Table 1). The results of APACHE III and SIRS scores for the same patient were all significantly correlated with their mortality, but the LODS, OASIS and SOFA scores was only weakly associated with mortality in patients with sepsis. We can infer from this that the APACHE III and SIRS scoring systems are able to predict the possibility of death from sepsis more accurately than the other three scoring systems. We also found that the higher the score, the more critical the patient’s condition and the higher the mortality.

**Table 1:** Distribution of death rate by organ dysfunction scoring systems.

Organ Dysfunction Scoring Systems	Alive (n = 8744)	Dead (n = 800)	Z	p-value
APACHE III	55 (40, 75)	61 (48, 81)	-7.643	0.000
LODS	6 (4, 9)	6 (4, 9)	-1.686	0.092
SIRS	3 (2, 4)	3 (2, 3)	-5.174	0.000
OASIS	36 (30, 42)	36 (30, 42)	-0.161	0.872
SOFA	7 (4, 10)	7 (4, 10)	-0.904	0.366

### 3.2. General clinical information

Table 2 provides a breakdown by various clinical indicators of the 800 sepsis cases who died in hospital. BMI, urine output in 24 hours, heart rate and admission body temperature were included in the significant factors in sepsis death. The results showed that patients with lower BMI, less urine output in 24 hours, slower heartbeats and lower body temperature had a higher mortality. The average BMI, urine output in 24 hours, heart rate and admission body temperature of these cases was 27.24, 1378ml, 103/min, and 37.1°C respectively.

**Table 2:** Distribution of death rate by general clinical information.

General Clinical Information	Alive (n = 8744)	Dead (n = 800)	Z/X2	p-value
Age	64 (53, 74)	65 (53, 75)	-1.237	0.216
Gender			0.000	0.995
Male	4852 (91.6%)	444 (8.4%)		
Female	3892 (91.6%)	356 (8.4%)		
BMI	28.83 (24.84, 34.22)	27.24 (23.06, 31.69)	-7.760	0.000
Urine output	1470 (850, 2330)	1378 (750, 2174)	-3.283	0.001
Heart rate	106 (92, 121)	103 (89, 119)	-2.947	0.003
Respiratory rate	28 (24, 33)	27 (24, 32)	-1.328	0.184
Temperature	37.4 (37.1, 38.0)	37.1 (36.9, 37.4)	-15.088	0.000

According to the results showed in Table 3, the mortality of patients with higher BMI was significantly lower than that of patients with low BMI, and the mortality of underweight patients (BMI<18.5) was the highest (22.8%). Compared with normal-weight patients (18.5≤BMI≤25), underweight patients (BMI<18.5) had 12% higher risk of death, while overweight (25<BMI≤30) and obese (BMI>30) patients had a 7.9% and 6.7% probability of death, respectively.

**Table 3:** Further analysis of general clinical information.

Death Factor	Groups	N	Death number	Mortality (%)
BMI	<18.5	167	38	22.8
	18.5–25	2401	260	10.8
	25–30	2901	230	7.9
	≥30	4075	272	6.7
Urine output	<400ml	1039	99	9.5
	400-2500ml	6468	552	8.5
	≥2500ml	2037	149	7.3
Temperature	<36	86	18	20.9
	36–37	2006	254	12.7
	37–38.5	6174	494	8.0
	≥38.5	1278	34	2.7

Looking at temperature, we found that the lower the patient’s body temperature, the higher the mortality. The total number of patients with a low temperature (<36°C) was 86, or 0.9% of all patients, but the mortality was extremely high at 20.9%. More than half of the patients’ temperatures were between 37°C-38.5°C, and the mortality of this group was 8%. Patients whose temperatures were above 38.5°C had a mortality of only 2.7%, which was about a seventh of that of patients with low temperatures.

Another important finding was that urine output in the first 24 hours after admission was strongly associated with mortality in patients with sepsis. The lower the patient’s urine output, the higher the mortality. If the patient’s urine output was less than 400ml within 24 hours of admission, the death rate was 9.5%.

### 3.3. Comorbidities

According to preliminary data analysis, comorbidities of congestive heart failure and liver disease were significantly associated with mortality in patients with sepsis, while myocardial infarct, cerebrovascular disease, chronic pulmonary disease, renal disease, peripheral vascular disease, dementia and other chronic diseases, peptic ulcer disease, diabetes, paraplegia, rheumatism and other diseases had no significant effect on mortality in sepsis patients.

As shown in Table 4, if a patient with sepsis has congestive heart failure, the probability of death may be 9.3%, which is higher than the probability of patients without congestive heart failure (7.9%), and if a patient with sepsis has liver disease, the odds of death may be 9.9% which is higher than the odd of death of patients without liver disease (8.1%). And also congestive heart failure was the most common chronic diseases that the sepsis patients have with a number of 3572 (37.44% of all sepsis patients).

**Table 4:** Distribution of death rate by comorbidities.

Comorbidities	Alive (n = 8744)	Dead (n = 800)	X2	P
Myocardial infarct			2.906	0.088
No	7185 (91.8%)	638 (8.2%)		
Yes	1559 (90.6%)	162 (9.4%)		
Congestive heart failure			5.813	0.016
No	5503 (92.1%)	469 (7.9%)		
Yes	3241 (90.7%)	331 (9.3%)		
Peripheral vascular disease			1.606	0.205
No	7614 (91.8%)	684 (8.2%)		
Yes	1130 (90.7%)	116 (9.3%)		
Cerebrovascular disease			0.000	0.991
No	7781 (91.6%)	712 (8.4%)		
Yes	963 (91.6%)	88 (8.4%)		
Dementia			3.001	0.083
No	8348 (91.7%)	753 (8.3%)		
Yes	396 (89.4%)	47 (10.6%)		
Chronic pulmonary disease			0.493	0.482
No	6094 (91.7%)	548 (8.3%)		
Yes	2650 (91.3%)	252 (8.7%)		
Rheumatic			1.305	0.253
No	8381 (91.7%)	760 (8.3%)		
Yes	363 (90.1%)	40 (9.9%)		
Peptic ulcer disease			2.589	0.108
No	8427 (91.7%)	762 (8.3%)		
Yes	317 (89.3%)	38 (10.7%)		
Liver disease			6.314	0.012
No	7228 (91.9%)	633 (8.1%)		
Yes	1516 (90.1%)	167 (9.9%)		
Diabetes			0.074	0.786
No	6344 (91.6%)	584 (8.4%)		
Yes	2400 (91.7%)	216 (8.3%)		
Paraplegia			0.023	0.879
No	8393 (91.6%)	767 (8.4%)		
Yes	351 (91.4%)	33 (8.6%)		
Renal disease			3.589	0.058
No	6414 (91.9%)	562 (8.1%)		
Yes	2330 (90.7%)	238 (9.3%)		

**Fig 1:**
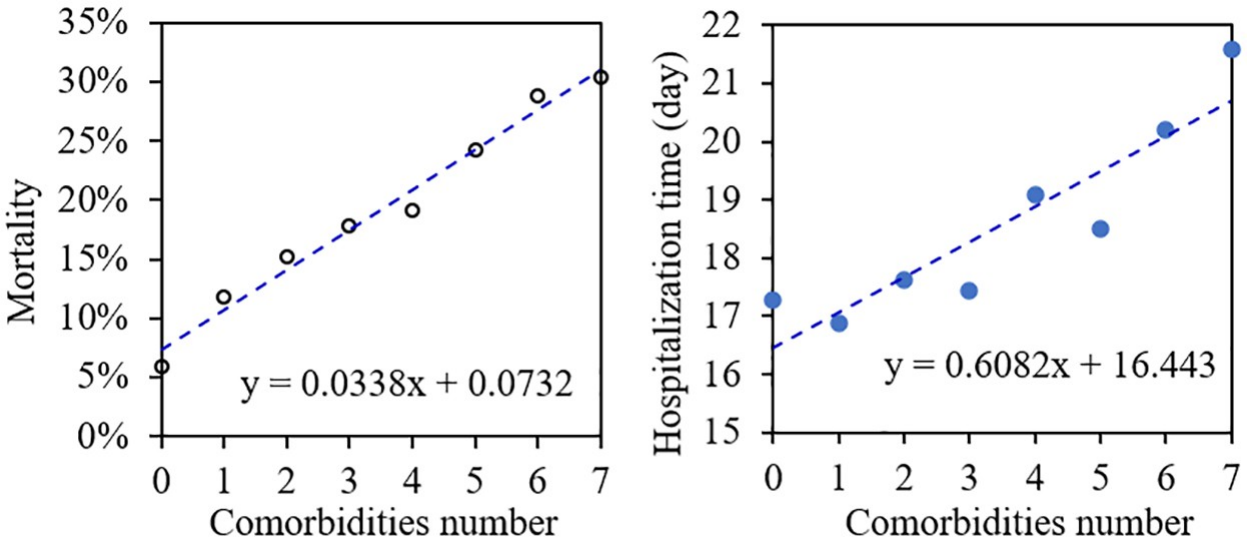
(a) the relationship between number of comorbidities and mortality-rate; (b) the relationship between number of comorbidities and the average time length spent in the hospital.

We also found that the more comorbidities the patient has, the greater the probability of death after developing sepsis (Fig 1). According to linear regression analysis, for each additional comorbidity in sepsis patients, the mortality increased by an average of 3.38%. When the patient has no underlying disease, the mortality rate after sepsis was about 7.32%. When the patient had one comorbidity, the mortality rate increased to 12%, and when the patient had more than 7 comorbidities, the mortality rate reached 30%. The number of comorbidities was also significantly related to length of hospitalization. For each additional comorbidity, the length of time spent in hospital increased by 0.6 days.

**Fig 2:**
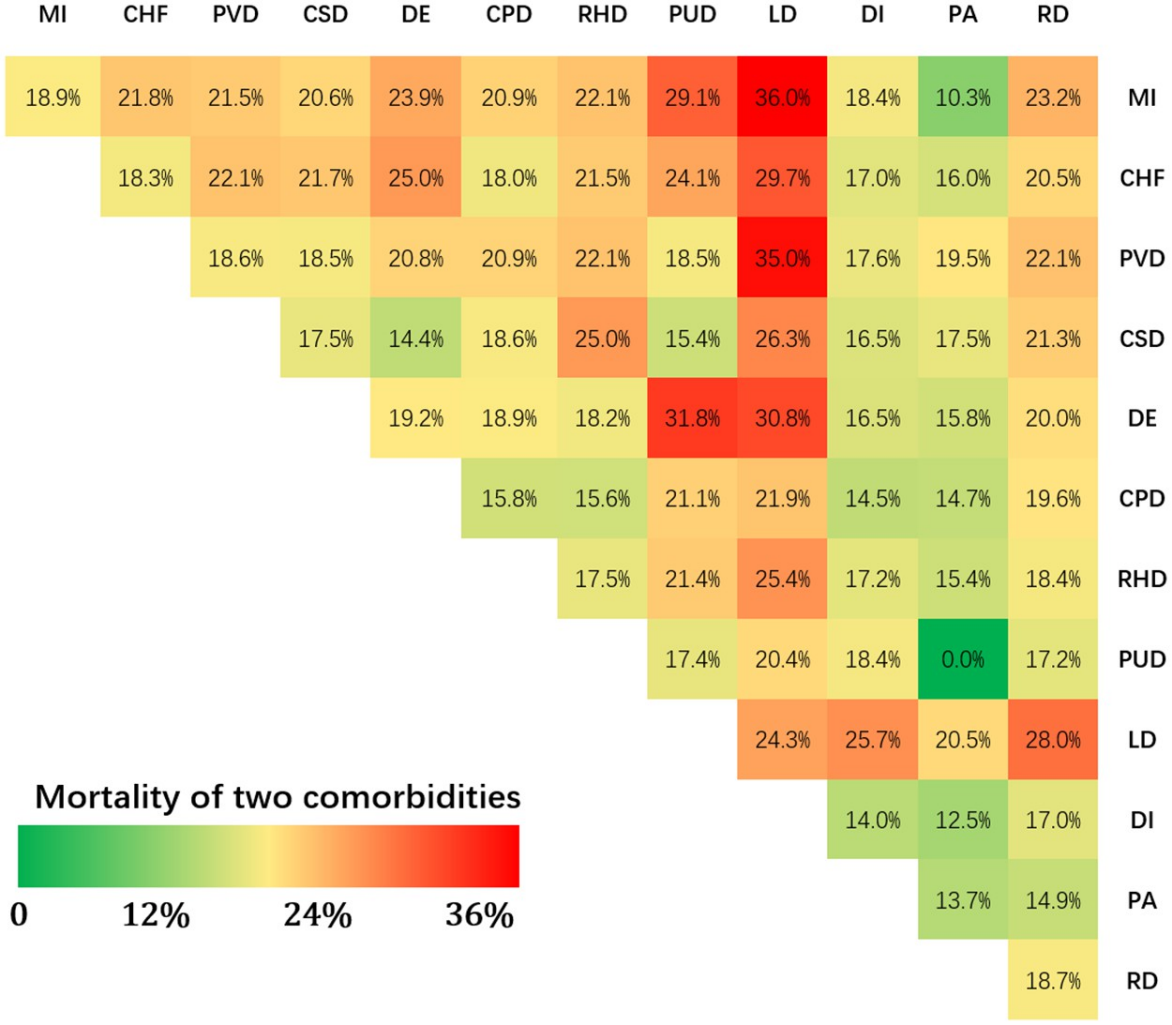
Relationship between mortality and each two comorbidities. (MI: myocardial infarction; CHF: congestive heart failure; PVD: peripheral vascular disease; CSD: cerebrovascular disease; DE: dementia; CPD: chronic pulmonary disease; RHD: rheumatic; PUD: peptic ulcer disease; LD: liver disease; DI: diabetes; PA: paraplegia; RD: renal disease).

To study the relationship between multiple comorbidities and mortality in patients with sepsis, we analyzed two comorbidities. After further analysis of the data of sepsis patients with two comorbidities, we obtained a relationship between mortality and comorbidities. As shown in Fig 2, in cases with liver disease and one other comorbidity, the average mortality rate in sepsis patients was higher than most of the other combination of two other comorbidities. For example, if a sepsis patient had only one comorbidity, for example a myocardial infarction, the probability of death would be 18.9%, however, if the same patient also had liver disease, the probability of death increased to 36.0%; the mortality of a sepsis patient only with a peripheral vascular disease was 18.6%, but the probability of death increased to 35.0% when the patient also had liver disease.

### 3.4. Multiple logistic regression analysis on factors affecting sepsis patients mortality

In order to further analyze the factors contributing to the death of sepsis patients, the significant indicators from the single factor analysis, which include APACHE III, SIRS, BMI, rine output, heart rate, temperature, congestive heart failure, and liver disease, were used as independent variables with patients status (alive/dead: 0/1) as the dependent variable. Stepwise (forward: LR) multiple logistic regression analysis was conducted. According to the results in Table 5, it was found that the independent factors affecting patients mortality were APACHE III, BMI, temperature, and liver disease (P <0.05). Among them, APACHE III (B: 0.008, OR: 1.008, 95%CI: 1.006–1.011, P: 0.000) and liver disease (B: 0.217, OR: 1.242, 95%, CI: 1.035–1.491, P: 0.020) were risk factors, which means that as the APACH III score increases and when there is liver disease, the likelihood of death increases. On the other hand, BMI (B: -0.043, OR: 0.958, 95%CI: 0.947–0.968, P: 0.000) and temperature (B: -0.722, OR: 0.486, 95%, CI: 0.438–0.539, P: 0.000) were protective factors, meaning that as BMI and temperature decrease, the likelihood of death also increases.

**Table 5:** Multiple logistic regression analysis.

	B	S.E.	Wald	df	Sig.	Exp(B)	95% C.I. for EXP(B)	
							Lower	Upper
APACHE III	0.008	0.001	37.570	1	0.000	1.008	1.006	1.011
BMI	-0.043	0.006	58.710	1	0.000	0.958	0.947	0.968
Temperature	-0.722	0.053	184.731	1	0.000	0.486	0.438	0.539
Liver disease	0.217	0.093	5.431	1	0.020	1.242	1.035	1.491

We listed the description statical analysis of all significant factors in Table 6.

**Table 6:** Description of significant factors.

	N	Average	Stand Variance	Median	Min	Max
APACHE III	9544	60.07	26.03	56	10	189
SIRS	9544	2.85	0.88	3	1	4
BMI	9544	30.26	8.26	28.7	14.67	84.63
Urine output	9544	1726.07	1292.61	1463.5	8	14415
Heart_rate	9544	107.33	21.34	106	53	199
Temperature	9544	37.55	0.81	37.4	32.6	42

## 4. Discussion

In 2020, the journal "Lancet" published a retrospective summary of sepsis patients around the world during 2017. There were about 48.9 million sepsis patients worldwide, of which about 11 million died, giving a case mortality rate of 19.7%, This estimate is 1.6 times the 31 million cases reported by Fleischmann et al. in 2016, indicating that the burden of sepsis worldwide has been severely underestimated. [13]. Also in 2020, "Critical Care Medicine" published China’s Sepsis data. Xie [14] conducted a study on sepsis in China’s ICUs based on this data. The results of that study show that 20% of ICU patients in mainland China are sepsis patients, and that the 90-day case mortality rate is as high as 35.5%, of which more than 60% of sepsis patients are patients with severe pneumonia. This also shows that Chinese patients with severe pulmonary infection account for a large proportion of sepsis patients.

### 4.1. Organ dysfunction scoring systems and sepsis mortality

According to the results of this study, the LODS, OASIS and SOFA scores were not significantly associated with mortality in sepsis patients, therefore, we focused on the analysis of the other two scoring systems.

In 1991, the American Thoracic Society and the American College of Critical Care Medicine held a joint meeting and proposed the diagnostic criteria for sepsis 1.0, namely the SIRS score. It mainly includes four items: body temperature >38°C or <36°C; heart rate > 90 bpm; respiratory rate >20 bpm or excessive ventilation, PaCO2 < 32mmHg; blood leukocyte count > 12 x 109/L or < 4 x 109/L (or immature granulocytes > 10%). When the infection and SIRS score≥2 is met, sepsis can be diagnosed [15]. Although sepsis 2.0 diagnostic criteria have been proposed in 2001, SIRS score has been applied to diagnose sepsis since its development. Due to the diagnostic requirements being too complex and lack of supportive evidence, sepsis 2.0 criteria have limited clinical application. Therefore, SIRS score should still be used in diagnosing sepsis [16]. Due to the SIRS diagnostic criteria, particularly the typical features of fever, tachycardia, and white blood cell changes that are indicative of infection, most infected patients meet SIRS criteria and are therefore considered to have sepsis. This method of defining sepsis results in a significant increase in the number of patients diagnosed with sepsis. However, the severity of their condition may be mild, thereby lowering the reported mortality rate [17]. Some studies have shown that the SIRS score is overly sensitive and lacks specificity [18, 19].

The Acute Physiology and Chronic Health Evaluation (APACHE) is the most widely used, extensively studied and far-reaching critical illness scoring system in clinical practice in the past 40 years. It has undergone continuous improvement and currently has four versions, including APACHE II and APACHE III, which are widely used in clinical applications. The APACHE I score was first proposed by Knaus et al. in 1981 and was composed of two parts: Acute Physiology Score (APS) and Chronic Health Score (CHS) before illness. Due to numerous indicators and the lack of consideration for the impact of age on prognosis, it is now less commonly used [20].

The APACHE III scoring system includes 17 physiological parameters which was developed in 1991 by Knaus et al. In comparison to the APACHE II score, six new variables were added to its acute physiology score (APS), which includes HCO3-, urea nitrogen, total bilirubin, blood glucose, serum albumin and urine output. The pH and PO2 were related to arterial blood gas analysis for scoring purposes. Additionally, each parameter was given a different scoring value and the CHS and age score were also refined and given higher values than those of the APACHE II score [21]. The severity of neurological conditions was evaluated by the patient’s ability to respond to pain or language stimulation by opening their eyes and showing movement changes, without using the GCS score [22]. This method was found to be more accurate in predicting patient prognosis and the risk of mortality in critical care patients. Studies have shown that APACHE III is superior to APACHE II in evaluating the severity and prognosis of critically ill patients [23].

It is known from statistics that there is a trade-off between sensitivity and specificity in the evaluation of sepsis, where pursuing higher sensitivity inevitably leads to lower specificity, requiring decision-makers to strike a balance. Currently, there are many scoring systems used in clinical practice for sepsis, and with the emergence of large electronic databases as well as the development of advanced algorithms such as machine learning and data mining, new scoring systems will continue to emerge, such as the APACHE III scoring system. Many studies have compared various scoring systems, but the patient populations and compared systems chosen in different studies vary, leading to different conclusions. Therefore, decision-makers need to weigh multiple indicators when choosing the optimal scoring system. In our study, there was no significant difference in predicting in-hospital mortality of sepsis patients between the SIRS and APACHE III systems. Accurately predicting the prognosis of ICU sepsis patients has significant clinical significance, and relies on an appropriate severity scoring system. However, defining and selecting an "appropriate" scoring system requires a comprehensive assessment of multiple studies and evaluation indicators. Therefore, we believe that using multiple scoring systems to cross-validate sepsis patients can help more accurately determine their severity and predict their prognosis.

### 4.2. General clinical information and sepsis mortality

The current research on the impact of sepsis on mortality of patients is still controversial [24]. According to the results of our previous multiple logistic regression analysis, only BMI and temperature are two factors that are significantly associated with mortality among the basic clinical information of patients with the p-values< 0.05, so we will conduct a detailed analysis of these two factors.

Obesity is an independent risk factor for death in the general population [25], and some studies have reported that being underweight is also associated with patient mortality, but these studies have not included BMI as an independent factor for clinical outcomes through multivariate analysis [26]. In contrast, Nguyen (2016) found that obese patients had a 16% lower risk of death compared with normal-weight hospitalized sepsis patients, according to an analysis of a large sample from more than 1,000 hospitals in the United States [27]. Some more in-depth analyses have shown that being overweight or obese (BMI ≥ 25.0) reduces mortality in ICU adults with sepsis, severe sepsis, or septic shock [28]. According to our analysis of the MIMIC IV sample data, the results of this study also support this finding: BMI is an independent factor of in-hospital death in sepsis patients, and obese sepsis patients with higher BMI have a lower mortality than normal-weight patients, while severely underweight (BMI <18.5) sepsis patients had the highest mortality.

The mechanism by which BMI is associated with sepsis mortality is unclear. First, sepsis involves an acutely abnormal metabolic state in which body fat can be used as energy in response to the body’s response to severe illness. Weight gain provides an indirect nutrient reserve that plays a critical role in life-threatening illnesses [29]. Studies have found that the positive effects of increased nutrition mainly occur in underweight patients, normal-weight patients and a small number of moderately obese patients, which indicate that attention to nutritional supplementation is particularly important for critically ill patients [30]. Second, higher BMI can lead to increased adipose tissue deposition. Studies have found that human macrophages can switch from M1 pro-inflammatory activation to alternative M2 anti-inflammatory activation during critical illness [31]. The protective effect of adipose tissue during critical illness may contribute more in obese individuals compared with healthy individuals of normal weight, therefore, obese patients with sepsis have less inflammation and tissue damage, less septic shock, and a higher survival rate. Third, increased adipose tissue is associated with enhanced activity of the renin-angiotensin system [32]. During sepsis, excessive fluid resuscitation and vasopressor use may be detrimental to critically ill patients [33, 34], but increased adipose tissue may have a hemodynamically protective effect and reduce the need for fluid or vasopressor support.

Fever (T>38°C), or hypothermia (T<36°C), are clinical manifestations of sepsis. Acute fever can account for more than 90% of all clinical manifestations in patients with septic infection, therefore, it is the most important clinical basis for diagnosing infection. Some frail or immunocompromised patients may have no symptoms of fever, some patients with severe infection may have hypothermia, and some patients may appear to have normal body temperature due to having taken antipyretics or physically cooled themselves before coming to the hospital. SIRS is an important intermediate link in the development of sepsis and was once one of the indicators for the diagnosis of sepsis. A change in body temperature is the primary diagnostic indicator of SIRS. Therefore, the possibility of acute infection should be considered in any patient with an elevated or abnormally low body temperature.

Fever is a regulated body temperature increase caused by the action of pyrogens that move the body temperature setpoint upward, and is the body’s response to foreign microbial infection. The effect of fever on the body has both advantages and disadvantages. A moderate increase in body temperature can inhibit the growth of bacteria, and enhance the immune function of the body by promoting the synthesis of autoantibodies and cytokines which can improve survival rate. In patients with sepsis, most patients have fever, but a small proportion (about 10%-20%) have hypothermia. Young et al. [35] believed that excessive body temperature elevation makes patients uncomfortable, increases the metabolic demands of the body, and causes damage to tissues and organs, ultimately increasing the risk of poor prognosis in patients. The results of Laupland et al. [36] also showed that compared with a body temperature <39.5 °C, patients with body temperature ≥39.5 °C were more likely to have arrhythmia, tachycardia, increased oxygen demand and even aggravated brain damage, and a significantly increased mortality. However, there is also some clinical research with findings inconsistent with the above results. In a study of rats with sepsis, hypothermia impaired the host’s immune defence response and increased infection complications compared with moderate fever [37]. The clinical research results of Yang et al. [38] also found that when the body temperature of patients with sepsis related fever, was cooled to between 36.0 °C and 37.5 °C, compared with cooling to 37.5 °C to 38.3 °C, the leukocytes of patients in the hypothermia group (36.0 °C to 37.5 °C) were significantly higher. The increased proportion of neutrophils suggests that hypothermia reduces the body’s ability to fight infection. A clinical study by Dias et al. [39] found that an increase in body temperature within the first 24 hours of admission to the ICU in infected patients was associated with a reduction in in-hospital mortality, and the patients with peak body temperature maintained at 39.0 °C to 39.4°C had the lowest risk of death. These results are consistent with what we found in this research: that the higher the patient’s body temperature within 24 hours of admission, the lower their mortality. We believe that the reasons are as follows: although low temperature can reduce the body’s inflammatory response by reducing the production of inflammatory factors, low temperature can also inhibit the migration of leukocytes and the phagocytic function of phagocytes, which will lead to aggravation of the body’s infection, coagulation dysfunction and other complications, all of which increase mortality.

### 4.3. Comorbidities and sepsis mortality

In addition to the patient’s basic clinical information, the patient’s own comorbidities often increase the risk of serious infection and considerably affect the survival rate of patients with sepsis. Studies have shown that infections are more common among alcoholics, malnourished persons, and people with chronic liver, kidney or cardiovascular disease, and patients with cancer or diabetes [40, 41]. Comorbidities may therefore increase the risk of infection, and sepsis patients with comorbidities have an increased risk of death. Charlson et al. proposed the CCI scoring system [42], which is an assessment tool for predicting the risk of death from disease by quantifying a patient’s comorbidities. The CCI score provides a reliable stratification method and includes 19 comorbidities, which are scored by disease weight, and the cumulative sum is the patient’s comorbidities score. However, in Charlson’s research, the sample size was generally small, and the mortality of sepsis patients with two or more diseases at the same time, was not studied in depth. In this study, we performed an in-depth analysis of the mortality of patients with sepsis by various comorbidities, the results are shown in Fig 2.

Since sepsis patients with liver disease had the highest mortality, we performed a further analysis of liver disease among all the comorbidities. The reason is that patients with poor liver function will develop jaundice, anemia, hemorrhage, adrenal insufficiency, hypoalbuminemia, and become malnourished. In addition they may develop portal hypertension such as ascites, formation of collateral circulation, and hypersplenism. In severe cases, complications such as gastrointestinal bleeding may occur [43]. Therefore, the worse the liver functioning is, especially when the patient also had another comorbidity with liver disease at the same time, the poorer is the body’s resistance, the more disordered the internal environment, and the more severe the condition after sepsis.

## 5. Conclusions

Sepsis progresses rapidly and can easily lead to severe organ dysfunction and septic shock. Early identification in critically ill patients, and timely transfer to ICU for treatment can reduce patient mortality. This study shows that the diagnosis of APACHE III should be completed in a timely manner in sepsis patients who are underweight, with low body temperature, low urine output, and two or more emergent disease comorbidities, especially liver disease. The severity of the case should be comprehensively assessed by combining SIRS scores. When the APACHE III score is greater than 60, the patient should be immediately transferred to the ICU to reduce the risk of death.

This study identified sepsis early based on a comprehensive evaluation of a patient’s basic information, organ dysfunction scores, and comorbidities, and the methodology could be used for actual clinical diagnosis in hospitals. However, this study has some limitations regarding the outcome measures used which only considered in-hospital mortality and did not take into account time factors such as commonly used indicators in clinical research such as 7-day mortality rate and 28-day mortality rate possibly leading to bias. In the future, more in-depth and detailed studies could be done by incorporating factors such as ICU treatment duration, 28-day mortality rate, and treatment methods into consideration.
